# Neonatal thyrotoxicosis secondary to maternal graves’ disease: a case report

**DOI:** 10.1186/s12887-026-06527-w

**Published:** 2026-01-21

**Authors:** Safa Elhassan, Ayman Abdalgader, Ahmed Gharib, Hossam Ahmed Mourad, Bashair Aldossari

**Affiliations:** 1 Department of Neonatal Intensive Care Units, Ministry of Health, Riyadh, Kingdom of Saudi Arabia; 2 Department of Clinical Pharmacy, Ministry of Health, Riyadh, Kingdom of Saudi Arabia

**Keywords:** Neonatal thyrotoxicosis, Graves’ disease, Neonatal intensive care unit, Antithyroid medications, Beta-blockers, Late-onset neonatal thyrotoxicosis

## Abstract

**Introduction:**

Neonatal thyrotoxicosis is a rare condition that often results from maternal antibody transfer, particularly in mothers who have had Graves’ disease. This disorder causes excessive thyroid hormone production in newborns. It can present with symptoms like tachycardia, poor weight gain, irritability, and, in severe cases, heart failure. Maintaining a high index of suspicion, making a prompt diagnosis, and intervening immediately are crucial. These steps help to mitigate the risk of long-term or serious complications.

**Case history:**

We present a neonate with late-onset neonatal thyrotoxicosis. The baby was born to a mother with Graves’ disease, who was on antithyroid medication and regular endocrinology follow-up. The maternal thyroid receptor antibody (TRAb) test was unavailable on either the day of delivery or the re-admission day. The infant was initially stable and discharged on the third day of life without concerns. On day 14, he presented with tachypnea and tachycardia, requiring neonatal intensive care unit admission. Thyroid function tests and symptoms confirmed late onset of thyrotoxicosis (TSH: 0.002 µIU/mL, FT4: 49.6 pmol/L). After the treatment with antithyroid medication and beta-blockers, symptoms and biochemical parameters improved markedly, allowing discharge after the stabilization. This case uniquely illustrates the diagnostic challenges associated with late-onset neonatal thyrotoxicosis when maternal TRAb screening is omitted, potentially exacerbating cardiac complications like mitral regurgitation-a feature less emphasized in prior reports.

**Conclusion:**

This case report highlights delayed-onset neonatal thyrotoxicosis complicated by mitral regurgitation, occurring in the context of absent maternal TRAb screening and early postnatal discharge. It demonstrates the masking effect of maternal antithyroid therapy and reveals deficiencies in anticipatory neonatal surveillance. This case expands the recognized spectrum of cardiac complications and emphasizes the importance of systematic maternal antibody screening. Further prospective studies are required to establish evidence-based surveillance protocols and cardiac risk stratification for neonates born to mothers with Graves’ disease.

## Introduction

Neonatal thyrotoxicosis is a rare disorder defined by excessive thyroid hormone production in newborns. Etiologies include the transplacental transfer of maternal thyroid-stimulating antibodies from women with autoimmune thyroid disease or abnormal fetal thyroid gland development [1, 2]. During the first trimester, fetal thyroid function is absent; therefore, maternal thyroid hormones, transferred via the placenta, are critical for normal fetal development. The proportion of maternal thyroid hormones crossing the placenta ranges from 20% to 50% [3]. Fetal thyroid function becomes fully established by 25 weeks of gestation [4]. The transplacental passage of thyroid-stimulating hormone receptor antibodies (TRAb) increases during the late second and third trimesters, coinciding with the period of maximal placental permeability and resulting in higher fetal serum concentrations. This process can induce neonatal thyrotoxicosis [5]. This condition arises primarily from the transplacental transfer of TRAb in women with Graves’ disease, leading to excessive fetal thyroid hormone production [3]. Graves’ hyperthyroidism affects approximately 0.2% of pregnant women, and neonatal Graves’ disease develops in 1% to 5% of their infants [6].

It is important to monitor TRAb serum levels in mothers with known cases of Graves’ disease before becoming pregnant, during and after pregnancy. The monitoring should continue even if she is euthyroid under anti-thyroid therapy or after thyroidectomy. Some studies have shown that fetal and neonatal thyrotoxicosis occurs only in women with three to five times the normal serum concentrations of stimulating TRAb [7]. Early-onset neonatal thyrotoxicosis refers to clinical and biochemical hyperthyroidism manifesting at birth or within the first 72 h of life, whereas late-onset cases occur after this period [8]. Late-onset forms account for a significant proportion of cases, particularly when mothers are euthyroid on therapy, as TRAb levels may remain elevated despite normal maternal thyroid function [9].

The clinical manifestations of neonatal hyperthyroidism are the same as those of hyperthyroidism in general [6, 10]. Diagnosis relies on maternal history, elevated neonatal free thyroxine (FT4) and suppressed thyroid-stimulating hormone (TSH), and confirmatory TRAb levels [11]. An elevated free triiodothyronine level is not required for the diagnosis of neonatal thyrotoxicosis, since affected neonates may exhibit suppressed TSH and isolated FT4 elevation, especially in the early period following maternal antithyroid drug exposure [12]. The onset and severity of symptoms vary, but they may depend on whether the mother takes antithyroid medication during delivery. Unlike early-onset cases, which may present at birth or within the first few days, late-onset thyrotoxicosis is frequently associated with maternal use of antithyroid drugs (e.g., methimazole or carbimazole) during pregnancy. These medications cross the placenta and suppress fetal thyroid function, masking symptoms until maternal drug levels clear from the neonatal circulation, a process that takes approximately 7–10 days [4, 5].

This case report presents a neonate with delayed thyrotoxicosis symptoms at the age of 14 days, highlighting the clinical presentation, diagnostic workup, and management of this condition. Prompt management with antithyroid drugs (e.g., methimazole equivalents) and beta-blockers is essential to prevent long-term sequelae like growth retardation or hyperactivity [13]. By sharing this case, we aim to increase awareness of neonatal thyrotoxicosis among healthcare providers and improve the recognition and treatment of this potentially life-threatening condition in newborns.

## Case history

A 14-day-old male infant was brought to the Emergency Room (ER) with tachypnea, mild subcostal recessions noted five days prior, and an audible systolic murmur. Chest radiography revealed cardiomegaly. The infant was delivered via emergency cesarean section at 38 weeks’ gestation due to failure to progress. There were no perinatal complications. Birth weight was 2.8 kg, and Apgar scores were 8 and 9 at one and five minutes of age, respectively. The 38-year- old mother was gravida 4, para 1 + 2 (G4 P1 + 2) with a documented history of oligohydramnios. She received 5 mg of oral Carbimazole daily for thyrotoxicosis, maintaining normal thyroid function throughout pregnancy. Measurement levels of TSH receptor-binding antibodies were not available during pregnancy or at the time of delivery.

The infant’s TSH level was within normal limits on the neonatal screening test performed after birth. Both the infant and mother remained in the postpartum care center for three days prior to discharge. A follow-up appointment was scheduled for the infant at seven days of age to undergo thyroid function tests (TFT) in the outpatient department (OPD); however, this appointment was not attended. Although the infant exhibited appropriate weight gain, he developed shortness of breath during and after feeding, with the symptoms progressively worsening.

**Fig. 1:**
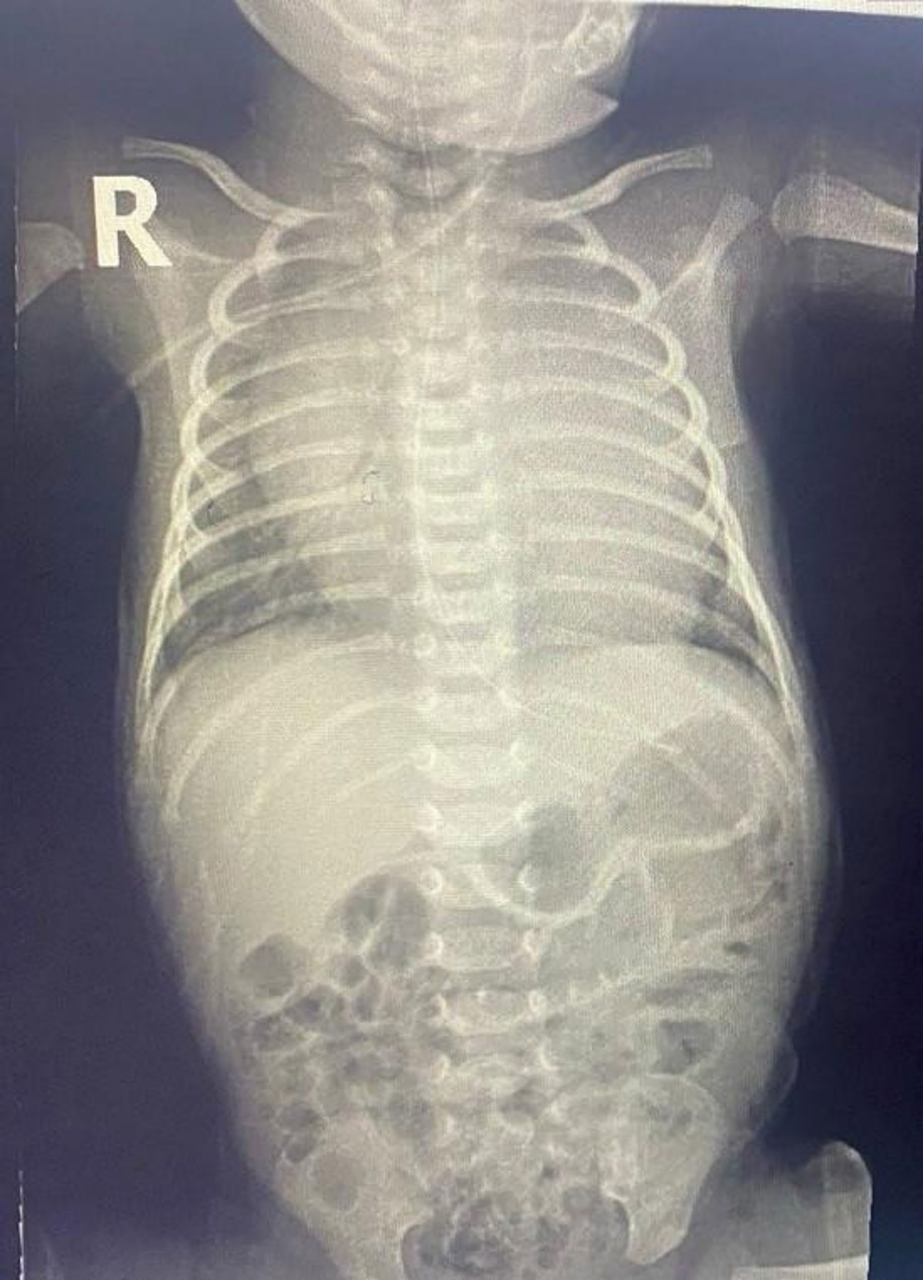
Thoracoabdominal babygram demonstrating cardiomegaly with an enlarged cardiac silhouette

At the time of admission, the patient exhibited tachypnea, retraction of the chest wall, tachycardia, an active precordium, a staring expression and mild lid lag. The neurologic exam showed jitteriness and irritability. His body weight was 3.150 kg (10th percentile), height was 52 cm (50th percentile), and head circumference was 33 cm (10th percentile). His blood pressure, pulse rate, respiratory rate, oxygen saturation, and body temperature were 85/41 mmHg, 180–200 bpm (sinus tachycardia), 70 breaths/min, 98%, and 36.8 °C, respectively. Initial laboratory findings were as follows: hemoglobin 16.2 g/dL, white blood cell count 18.7 × 10^9^/𝐿, platelet count 372 × 10^9^/𝐿, red blood glucose 97 mg/dL, aspartate transaminase (AST) 32 IU/L, alanine transaminase (ALT) 30 IU/L, total bilirubin 6.6 umol/L, albumin 28.4 g/L, gamma-glutamyl transferase (GGT) 165 IU/L, serum creatinine 25.8 umol/L, calcium level 2.43 mmol/L, sodium level 138 mmol/L, potassium level 5.9 mmol/L, Magnesium level 0.79 mmol/L and phosphate level 2.43 mmol/L. Thyroid function test revealed elevated free thyroxine (FT4) 49.6 pmol/L (reference range 10.3–25.8 pmol/L), normal free triiodothyronine (FT3) 8.75 pmol/L (reference range 3 −9.28 pmol/L), and decreased TSH 0.002 µIU/mL (reference range 0.72 −11.0 uIU/mL). Tests for TRAB, thyroid peroxidase, and thyroglobulin antibodies was unavailable. C-reactive protein (CRP) tests conducted on two separate occasions were negative. Furthermore, both blood cultures and Methicillin-Resistant Staphylococcus Aureus (MRSA) tests were also negative. The metabolic screening performed at 24 h of age yielded normal result. Chest radiography revealed cardiomegaly in the anteroposterior view (see Fig. 1). Echocardiography (ECHO) identified a small 3 mm patent foramen ovale (PFO) with a left-to-right shunt. The ventricular septum was intact and there was no evidence of patent ductus arteriosus (PDA). Mild mitral regurgitation (MR) was observed, with a pressure gradient of 30 mmHg. The atrioventricular (AV) and pulmonary valves (PV) were normal, as were systemic and pulmonary venous return. Systolic function was preserved, with an ejection fraction (EF) of 73%. No pericardial effusion was identified. Repeating ECHO on day 18 of life showed resolution of mitral regurgitation. Thyroid ultrasound was not performed, as current guidelines do not strongly recommend its use in neonates.

Oral Carbimazole was initiated at a dose of 750 mcg/kg/day divided into every 8 h (Methimazole was unavailable), in conjunction with oral Propranolol at 2 mg/kg/day divided every 8-hours. Twenty-four hours post-admission, the patient exhibited persistent shallow, rapid respiration (respiratory rate 70 beaths/min) and tachycardia (heart rate 200 beats/min) accompained by subcostal (SC) and intercostal (IC) retractions. Capillary blood gas (CBG) analysis showed: pH 7.45, PCO_2_ 33mmHg, HCO_3_ 22.9mmol/L, and base excess (BE) −1.1mmol/L. After one week of treatment, thyroid function improved as demonstrated by FT4 (26.7 pmol/L), FT3 (8.75 pmol/L), and TSH < 0.005 µIU/mL. Based on these improvement, the endocrinologist advised continuing the current regimen and repeating the thyroid function tests weekly. The patient received oxygen by nasal cannula for about 10 days, transitioning to room air at 24 days old after resolution of tachypnea due to neonatal thyrotoxicosis. Thyroid hormone levels continued to improve, with FT4 (16.7 pmol/L), FT3 (7.6 pmol/L), and TSH 0.015 µIU/mL on day 15 of hospitalization.

At 36 days of age (hospital day 22), the patient’s heart rate remained within normal limits. The oral propranolol dosage was reduced to 1.3 mg/kg/day, administered every 12 h, and discontinued on hospital day 27. Thyroid function continued to improve, with FT4 at 14.3 pmol/L, FT3 at 6.02 pmol/L, and TSH at 0.112 µIU/mL.

At 42 days of age, the TRAb level was 25.42 IU/L, which is significantly above the reference range of 0 to 2 IU/L. On the same day, thyroid function tests continued to improve with FT4 at 17.1 pmol/L, FT3 at 6 pmol/L, and TSH at 0.230 µIU/mL. As these improvements in laboratory results, the baby’s general condition gradually stabilized. Consequently, he was discharged on day 42 of life (after 28 days in the hospital) on oral Carbimazole, at a dosage of 750 mcg/kg/day divided every 8 h. The patient was referred to an endocrinologist for further management and treatment in an outpatient care setting.

### Post-discharge follow-up

**Fig. 2:**
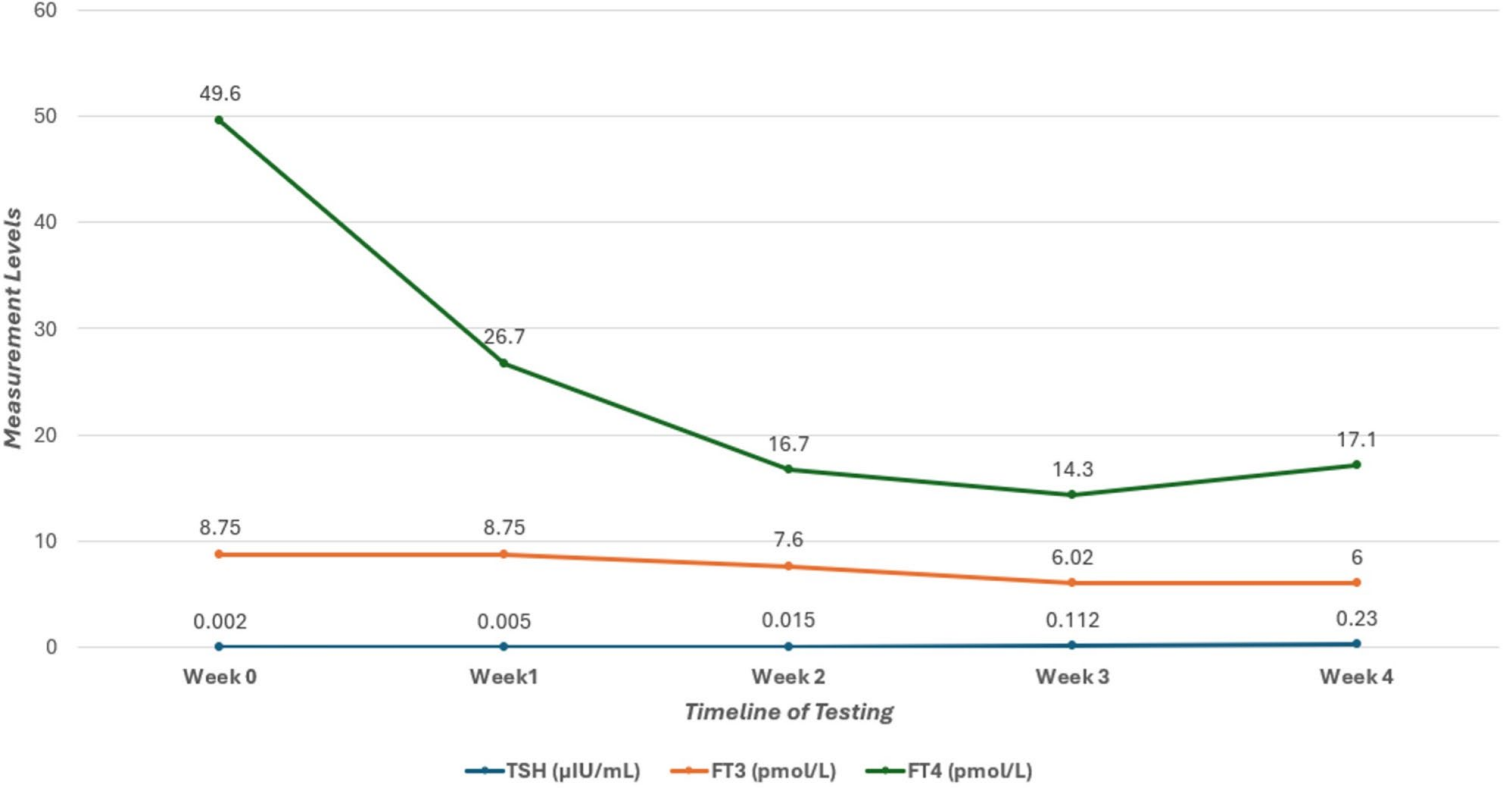
Sequential thyroid function test results were recorded throughout the hospitalization period

The infant had regular followed up with outpatient endocrinology clinic. The thyroid function tests remained within the normal range and TRAb levels declined over time. Consequently, carbimazole was gradually tapered and discontinued at 6 months of age, with no recurrence of symptoms. At 6 months, repeat TFTs confirmed continued normal results, and echocardiography demonstrated no residual cardiac abnormalites. Additionally, no complications, including developmental delays or hyperactivity were observed, which is consistent with timely intervention. Following these favorable outcomes, the patient was discharged from the endocrinology clinic, and the family was advised to seek medical attention if any thyroid-related symptoms emerged fig. 2. 

## Discussion

This case report describes a neonate diagnosed with thyrotoxicosis who exhibited symptoms on the 14th day of life, including irritability, tachycardia and tachypnea. ECHO showed mild mitral regurgitation which is one of rare complications of neonatal thyrotoxicosis. Mitral regurgitation in this context likely reflects transient thyrotoxic myocardial dysfunction rather than structural heart disease. The differential diagnosis for these clinical manifestations includes sepsis and congenital heart disease. However, given the relevant maternal history, neonatal thyrotoxicosis secondary to Graves’ disease should also be considered. The mother, previously diagnosed with Graves’ disease, received oral Carbimazole at a dose of 5 mg daily and maintained euthyroid status throughout pregnancy. Documentation of thyroid-stimulating hormone receptor antibody levels was unavailable for his mother. Diagnostic confirmation was based on elevated free thyroxine (FT4), normalized free triiodothyronine (FT3), suppressed thyroid-stimulating hormone (TSH), and elevated TSH receptor antibodies (TRAb) measured at 14 days of age. The TRAb test was requested at the time of admission; however, this assay is not available at the reporting facility. The patient exhibited a normal FT3 level. This finding can be attributed to the immature peripheral deiodinase activity in neonates, which limits the conversion of FT4 to FT3 [8, 12]. Additionally, maternal antithyroid therapy may preferentially suppress FT3 production [8, 12]. Normalization of FT3 levels does not exclude the possibility of thyrotoxicosis.

This case shares key features with previously reported instances of neonatal thyrotoxicosis, while also emphasizing unique risks associated with omissions in prenatal screening. Fine et al. (2024) reported a case in which the mother had a documented history of hyperthyroidism, and the newborn presented with neonatal hyperthyroidism accompanied by hypoglycemia and other clinical manifestations. Laboratory tests revealed elevated thyroid-stimulating immunoglobulins (TSIs), confirming the diagnosis of neonatal hyperthyroidism; however, unlike our case, prenatal TRAb monitoring was documented, allowing earlier intervention and avoiding cardiac complications like mitral regurgitation [14]. Similarly, in our case, the mother had a well-documented history of thyrotoxicosis and was undergoing treatment with oral Carbimazole during pregnancy. The newborn exhibited elevated TRAb and was subsequently diagnosed with neonatal hyperthyroidism, consistent with previously reported cases.

Another relevant case report by Zhu et al. (2024). described a neonate diagnosed with hyperthyroidism born to a mother with Graves’ disease. The diagnosis in their case was based on the criteria outlined in the Practice of Neonatology (5th Edition), which include: (1) a maternal history of autoimmune thyroid disease, particularly hyperthyroidism; (2) the presence of typical signs and symptoms of neonatal hyperthyroidism; and (3) supportive laboratory findings, including elevated serum T3 and T4 levels and suppressed TSH levels. As in Zhu et al.‘s report (2024), the neonate in the present case met al.l these diagnostic criteria, which further supports the diagnosis of neonatal hyperthyroidism [15].

Based on the novel insights and identified gaps in clinical practice, we compared our case with those reported by Fine et al. (2024) and Zhu et al. (2024) [14, 15]. Unlike cases with documented prenatal TRAb monitoring, our infant’s delayed presentation highlights the need for standardized neonatal screening protocols in high-risk births: consistent with American Thyroid Association (ATA) recommendations to prevent avoidable complications such as transient mitral regurgitation [14, 15]. The delayed presentation on day 14 and the occurrence of transient mitral regurgitation with pressure gradient of 30 mmHg in our case further demonstrate the increased morbidity risk associated with the absence of standardized screening, as advocated by the American Thyroid Association (ATA) guidelines [16].

The global prevalence of hyperthyroidism during pregnancy is estimated at 0.7% to 2.8% [14]. Neonatal hyperthyroidism is a rare but potentially life-threatening condition, occurring in approximately 1 in 50,000 live births [17]. This condition most often results from the transplacental passage of maternal thyroid-stimulating immunoglobulins (TSIs or TRAb), which activate fetal TSH receptors and consequently lead to excess thyroid hormone production [18, 19].

Antibodies may persist in mothers who are clinically euthyroid following treatment (either with medication, surgery, or radioactive iodine) and these antibodies can adversely affect the fetus or neonate. Consequently, neonatal hyperthyroidism remains a risk for infants born to mothers with a history of Graves’ disease if circulating TRAb are present [10]. In clinical practice, TRAb measurement is often omitted, particularly in asymptomatic or euthyroid mothers [10, 20]. This omission complicates early identification and risk stratification of affected neonates. Van Der Kaay et al. (2016) emphasized that TRAb can persist in euthyroid mothers’ post-treatment, thereby increasing fetal risk—a direct parallel to our maternal profile—and recommended monitoring to prevent complications, which our case lacked, leading to avoidable delays [21]. Unlike these studies, this report uniquely links omitted screening to transient mitral regurgitation observed in the presented neonatal case. This cardiac sequela is not prominently detailed in prior neonatal thyrotoxicosis literature. The present finding addresses a critical gap in understanding preventable outcomes.

The 2016 American Thyroid Association guidelines recommend screening for TRAb in all pregnant women with a history of Graves’ disease during the first trimester. If the test is positive, it should be repeated at 18–22 and 30–34 weeks’ gestation. TRAb levels greater than or equal to 5 IU/L, or three times the upper limit of normal, increase the risk of fetal or neonatal thyrotoxicosis. In such cases, fetal thyroid ultrasound is also recommended [22].

Neonatal thyrotoxicosis can present with nonspecific symptoms including tachycardia, irritability, tachypnea, thrombocytopenia, hepatomegaly, jaundice, hypoglycemia, hyperhidrosis, goiter, exophthalmos, pulmonary hypertension, and intrauterine growth restriction [1]. In severe cases, it leads to heart failure, neurodevelopmental delay, or death [1]. The onset and severity of symptoms depend on the maternal antibody titers, control of maternal thyroid status, and neonatal metabolism. Early diagnosis and providing proper management are challenges faced by each healthcare provider. Any delay in diagnosis or providing appropriate management can lead to serious complications such as growth retardation, craniosynostosis, hyperactivity, developmental and behavioral problems and permanent central hypothyroidism [6, 10]. Such studies recommended that before starting therapy, the total triiodothyronine (T3) concentration, FT4, and TSH levels should be measured as a baseline for monitoring therapy [6]. They recommend treating with the first line of therapy, which is Methimazole and a beta-adrenergic blocker (Propranolol) for these neonates after confirming the diagnosis [6]. Samuels et al. (2018) highlighted nonspecific symptoms like tachycardia and irritability, with risks of heart failure if untreated, comparable to our patient’s initial presentation but mitigated by timely beta-blocker use [17].

In this case, the neonate presented on day 14 with irritability, tachypnea, tachycardia, and a heart murmur. Such presentations are frequently misdiagnosed as neonatal sepsis, congenital heart disease, or substance withdrawal, which may delay appropriate diagnosis and medical interventions. Therefore, a high index of suspicion is essential in neonates born to mothers with a history of autoimmune thyroid disease. A thorough clinical assessment and a stepwise diagnostic approach enabled a timely diagnosis in our case, followed by the prompt initiation of therapy. Treatment was initiated with oral carbimazole and propranolol. Carbimazole was administered at 750 mcg/kg/day devided into every 8 h (substituted for methimazole, which was unavailable), using a 10 mg: 6 mg carbimazole-to-methimazole conversion ratio as described by Bohîlțea et al. (2022), with interpretation guided by neonatal reference ranges from Ogilvy-Stuart (2002) [1, 2]. Propranolol was initiated concurrently at 2 mg/kg/day, divided into 8-hourly doses. Thyroid function was monitored regularly to guide treatment adjustment.

Supportive care—such as respiratory and circulatory support in cases with cardiac or pulmonary involvement—is crucial. Sufficient caloric intake plays an important role in supporting neonatal growth. In some studies, short-term glucocorticoids (e.g., hydrocortisone 2.5–10 mg/kg/day or prednisone 1–2 mg/kg/day) have been used to suppress thyroid hormone secretion and peripheral T4-to-T3 conversion [14, 20]. In severe or resistant cases, intravenous immunoglobulin (IVIG) has also been utilized [13].

As thyrotropin receptor antibody (TRAb) levels decline, clinical manifestations of neonatal thyrotoxicosis typically resolve. Antithyroid therapy can be discontinued once TRAb levels are undetectable [11]. The American Academy of Pediatrics (AAP) recommends administering antithyroid drugs and beta-blockers as needed, accompanied by close clinical monitoring until at least three months of age [16]. Timely initiation of appropriate therapy generally results in rapid clinical improvement in most neonates with hyperthyroidism. Nevertheless, some children exhibit intelligence quotients in the 80 s at school age, even after receiving prompt neonatal treatment [6].

The neonate demonstrated a favorable response to treatment and was discharged after 28 days with normalized thyroid function, as shown in Table No. 1. Upon discharge, oral Carbimazole was prescribed at a dosage of 750 mcg/kg/day, and an endocrinology follow-up was scheduled at the outpatient clinic. Neonatal hyperthyroidism is generally transient, resolving within 3 to 12 weeks [21]. However, mortality rates in severe, untreated cases may reach 15 to 20% [16].

**Table 1:** Sequential Thyroid Function Tests and Pharmacological Interventions in a Neonate with Late-Onset Thyrotoxicosis

Time point (weeks from admission)	TSH (μIU/mL)	FT3 (pmol/L)	FT4 (pmol/L)	TRAb (IU/L)	Therapy regimen
Baseline (week 0)	0.002	8.75	49.6	Not available	Carbimazole 750 mcg/kg/day divided into every 8 hours, equivalent to 0.45 mg/kg/day methimazole*) & Propranolol 2 mg/kg/day orally divided every 8 hours.
Week 1	<0.005	8.75	26.7	Not available	Continued carbimazole and propranolol
Week 2	0.015	7.6	16.7	Not available	Carbimazole continued; Propranolol reduced by 35% to 1.3 mg/kg/day divided every 12 hours due to heart rate normalization.
Week 3	0.112	6.02	14.3	Not available	Carbimazole continued; Propranolol discontinued.
Week 4	0.23	6	17.1	25.42	Carbimazole was continued at a dose of 750 mcg/kg/day divided every 8 hours.

*Carbimazole-to-methimazole conversion ratio of 10mg:6mg, as per Bohîlțea et al. (2022) [1]

Reference ranges: TSH 0.72–11.0 μIU/mL, FT3 3–9.28 pmol/L, FT4 10.3–25.8 pmol/L, TRAb 0–2 IU/L. All measurements are performed via standard immunoassay in a clinical laboratory

### Limitations

Limitations include the delayed availability of neonatal TRAb and antibody tests, hindering immediate confirmation, and the retrospective maternal data. As standard of care TRAb screening for all neonates born to mothers’ with known case of graves’ disease to avert complications. In summary, this case, comparable to prior studies yet distinguished by its cardiac emphasis, advocates for routine prenatal TRAb assessment in mothers with Graves’ disease to enhance early detection and outcomes in neonatal thyrotoxicosis.

## Conclusion

This case uniquely demonstrates late-onset neonatal thyrotoxicosis with clinically significant cardiac involvement in an infant who was initially asymptomatic and discharged early, despite maternal Graves’ disease and unavailable thyroid receptor antibody status. The delayed presentation underscores that maternal antithyroid therapy may mask neonatal disease and create false reassurance during the immediate postnatal period. This report contributes to the literature by highlighting mitral regurgitation as a potentially underrecognized cardiac manifestation of delayed neonatal thyrotoxicosis and by illustrating the consequences of omitting maternal TRAb screening at delivery. These findings support the implementation of standardized, risk-based postnatal surveillance protocols, including mandatory maternal TRAb assessment and prolonged clinical monitoring of high-risk neonates. Further research should define optimal monitoring duration, evaluate cardiac outcomes in late-onset cases, and determine whether extended inpatient observation reduces morbidity in this population.

## Data Availability

No datasets were generated or analysed during the current study.

## Abbreviations

AAP: American Academy of Pediatrics
ALT: Alanine Transaminase
AST: Aspartate Transaminase
ATA: American Thyroid Association
CBG: Capillary Blood Gas
CRP: C-reactive protein
EBM: Expressed Breast Milk
ECHO: Echocardiogram
EF: Ejection Fraction
ER: Emergency Room
FT4: Free Thyroxine
G: Gravida
GGT: Gamma-Glutamyl Transferase
GNAS: Guanine Nucleotide-Binding Protein Alpha Stimulating
HFNC: High-Flow Nasal Cannula
IC: Intercostal
IUGR: Intrauterine Growth Restriction
IVIG: Intravenous Immunoglobulin
LFNC: Low-Flow Nasal Cannula
MR: Mitral Regurgitation
MRSA: Methicillin-Resistant Staphylococcus Aureus
NICU: Neonatal Intensive Care Unit
OPD: Outpatient Department
P: Parity
PDA: Patent Ductus Arteriosus
PFO: Foramen Ovale
SC: Subcostal
T3: Triiodothyronine
TFT: Thyroid Function Tests
TR: Tricuspid Regurgitation
TRAb: Thyroid Stimulating Hormones Receptor Antibodies
TSH: Thyroid Stimulating Hormones
TSHR: Thyroid-Stimulating Hormone Receptor
TSIs: Thyroid-Stimulating Immunoglobulins
WBCs: White Blood Cells
